# Supplementary material for: Second breast cancer following negative breast MRI: Analysis by interval from surgery and risk factors
Source: PLoS One. 2024 Aug 15;19(8):e0306828. doi: 10.1371/journal.pone.0306828 (PMC11326552; doi:10.1371/journal.pone.0306828)
Supplement: S1 Appendix — (DOCX) [file pone.0306828.s001.docx]

**S1 Appendix. Site of local-regional recurrences.**

The sites of local-regional recurrence were as follows: ipsilateral chest wall (n = 4), ipsilateral chest wall and supraclavicular lymph node (n = 1), ipsilateral axilla (n = 4), ipsilateral supraclavicular lymph node (n = 2), ipsilateral axilla and supraclavicular lymph node (n = 1), and ipsilateral infraclavicular lymph node (n = 1).
