# Supplementary material for: Second breast cancer following negative breast MRI: Analysis by interval from surgery and risk factors
Source: PLoS One. 2024 Aug 15;19(8):e0306828. doi: 10.1371/journal.pone.0306828 (PMC11326552; doi:10.1371/journal.pone.0306828)
Supplement: S1 Table — (DOCX) [file pone.0306828.s002.docx]

**S1 Table. Clinical-pathologic imaging characteristics of the 25 second breast cancers.**

|  |  |  | Characteristics of second breast cancers | | | | | | | | | | | | |
| --- | --- | --- | --- | --- | --- | --- | --- | --- | --- | --- | --- | --- | --- | --- | --- |
| Patient  age (y)* | Original  breast  cancer  histologic  type | Original  breast  cancer  hormonal  status | Initial  surgery  interval  (mo)† | MRI  interval  (mo)‡ | Initial surgery-MRI interval (mo) | Last US  interval  (mo)§ | Last  MG  interval  (mo)\|\| | Breast  site | Detection  modality | Visible  at MG | Symptoms | Histologic  type | Size  (cm)# | Nodal  status | Hormonal  status |
| 54 | DCIS | ER+, PR+ | 72.1 | 40.7 | 31.4 | 19.6 | 0.7 | Contra | US | No | No | DCIS | 0.3 | Neg | ER+, PR+ ** |
| 49 | IDC | ER+, PR-, HER2- | 51.5 | 26.8 | 24.7 | 6.3 | 14.2 | Contra | MG, US | Yes | No | IDC | 1.0 | Neg | ER-,  PR-, HER2- |
| 51 | IDC | ER+, PR-, HER2+ | 56.9 | 32.7 | 24.2 | 12.1 | 6.3 | Contra | MRI | No | No | DCIS | 1.4 | Neg | ER-,  PR- ** |
| 43 | DCIS | ER+, PR+, HER2- | 46.5 | 13.3 | 33.2 | 6.3 | 6.3 | Ipsi | MG, US | Yes | Yes | DCIS with MI | 0.1 | Neg | ER+, PR+, HER2- |
| 34 | IDC | ER+, PR+, HER2- | 106.6 | 70.9 | 35.7 | 13.0 | 13.0 | Ipsi | MG, US | Yes | No | IDC | 1.9 | Neg | ER+, PR+, HER2- |
| 31 | IDC | ER+, PR-, HER2- | 71.4 | 44.8 | 26.6 | 18.4 | 18.4 | Contra | US | No | No | IDC | 0.2 | Neg | ER-,  PR-, HER2- |
| 41 | DCIS | ER+, PR+ | 65.4 | 39.3 | 26.1 | 8.1 | 14.4 | Contra | MG, US | Yes | No | IDC | 3.0 | Pos | ER-,  PR-, HER2+ |
| 53 | IDC | ER-,  PR- | 97.2 | 73.0 | 24.2 | 12.6 | 12.6 | Ipsi | MG, US | Yes | No | IDC | 0.8 | Neg | ER-,  PR-, HER2- |
| 37 | IDC | ER+, PR+, HER2- | 52.3 | 28.2 | 24.1 | 4.9 | 4.9 | Contra | MRI | No | No | DCIS | 0.3 | Neg | ER+, PR+, HER2- |
| 48 | IDC | ER-,  PR-, HER2+ | 59.6 | 33.6 | 26 | 13.9 | 13.9 | Contra | MRI | Yes | No | IDC | 0.25 | Neg | ER+, PR+, HER2- |
| 49 | IDC | ER-,  PR-, HER2- | 84.7 | 54.5 | 30.2 | 12.1 | 12.2 | Contra | US | No | No | IDC | 0.8 | Neg | ER+, PR-, HER2- |
| 39 | DCIS | ER-,  PR-, HER2+ | 59.7 | 28.1 | 31.6 | 1.5 | 10.0 | Ipsi | MRI | Yes | No | IDC | 0.6 | Neg | ER+, PR+, HER2- |
| 57 | IMC | ER+, PR-, HER2+ | 54.0 | 30.8 | 23.2 | 12.0 | 12.2 | Contra | MG, MRI | Yes | No | IDC | 2.4 | Pos | ER+, PR-, HER2- |
| 47 | IDC | ER+, PR-, HER2- | 92.8 | 55.5 | 37.3 | 12.8 | 12.8 | Contra | MRI | No | No | IDC | 1.8 | Neg | ER+, PR-, HER2- |
| 50 | DCIS | ER+, PR+, HER2+ | 129.0 | 72.8 | 56.2 | 40.7 | 40.7 | Contra | US | Yes | No | DCIS | N/A | Neg | ER-,  PR-, HER2+ |
| 39 | IDC | ER+, PR-, HER2- | 84.0 | 41.0 | 43 | 16.4 | 28.7 | Ipsi | MG, US | Yes | No | IDC | 1.9 | Neg | ER+, PR-, HER2- |
| 49 | ILC | ER+, PR-, HER2- | 72.6 | 12.5 | 60.1 | 16.6 | 12.5 | Contra | US | No | No | DCIS | 0.4 | Neg | ER+, PR+, HER2- |
| 55 | IDC | ER+, PR-, HER2- | 85.0 | 48.9 | 36.1 | 21.4 | 28.1 | Ipsi | MG, US | Yes | No | DCIS | 0.8 | Neg | ER-,  PR-, HER2- |
| 25 | IDC | ER+, PR+, HER2- | 224.7 | 51.9 | 172.8 | 7.3 | 17.3 | Contra | US, MRI | No | No | IDC | 0.9 | Neg | ER+, PR-, HER2- |
| 49 | IDC | ER+, PR+, HER2+ | 89.6 | 32.3 | 57.3 | 13.3 | 13.3 | Contra | MG, US | Yes | No | DCIS | 0.9 | Neg | ER+, PR-, HER2+ |
| 55 | IDC | ER-,  PR-, HER2- | 120.2 | 64.6 | 55.6 | 12.6 | 12.6 | Contra | MG, US | Yes | No | IDC | 2.0 | Neg | ER-,  PR-, HER2- |
| 63 | IDC | ER+, PR+, HER2- | 107.0 | 50.9 | 56.1 | 13.3 | 13.3 | Contra | MG, US | Yes | No | IDC | 0.3 | Neg | ER-,  PR-, HER2- |
| 41 | IDC | ER+, PR+, HER2- | 81.1 | 31.6 | 49.5 | 12.7 | 12.7 | Contra | US | No | No | IDC | 0.4 | Neg | ER+, PR+, HER2- |
| 66 | IDC | ER+, PR+, HER2- | 85.8 | 42.4 | 43.4 | 5.1 | 5.1 | Ipsi | US | No | No | IDC | 0.9 | Neg | ER+, PR+, HER2- |
| 43 | DCIS | ER+, PR-, HER2+ | 59.9 | 35.2 | 24.7 | 11.1 | 11.1 | Ipsi | MRI | No | No | IDC | 0.8 | Neg | ER+, PR+, HER2+ |
| Note.—Contra = contralateral breast, DCIS = ductal carcinoma in situ, ER = estrogen receptor, ER- = ER-negative, ER+ = ER-positive, HER2 = human epidermal growth factor receptor type 2, HER2- = HER2-negative, HER2+ = HER2-positive, IDC = invasive ductal carcinoma, IMC = invasive micropapillary carcinoma, ILC = invasive lobular carcinoma, Ipsi = ipsilateral breast, MG = mammography, MI = microinvasion, Neg = negative, Pos = positive, PR = progesterone receptor, PR- = PR-negative, PR+ = PR-positive, N/A = not available  * Age at original breast cancer diagnosis.  † Interval between original breast cancer surgery and detection of second in-breast cancer.  ‡ Interval between screening MRI and detection of second in-breast cancer.  § Interval between last US preceding detection of second in-breast cancer and detection of second in-breast cancer.  \|\| Interval between last mammographic examination preceding detection of second in-breast cancer and detection of second in-breast cancer.  # Pathologic size of invasive cancer component only for IDC or DCIS with microinvasion or pathologic size of DCIS component for pure DCIS. However, this is the pretreatment imaging-based size for the 3.0-cm, node-positive IDC case, as the patient underwent neoadjuvant chemotherapy and achieved pathologic complete response.  The imaging-based size is also given for the 0.8-cm IDC case in a 43-year-old patient, as she received surgery at a different institution.  ** HER2 status was missing. | | | | | | | | | | | | | | | |
