# Supplementary material for: Second breast cancer following negative breast MRI: Analysis by interval from surgery and risk factors
Source: PLoS One. 2024 Aug 15;19(8):e0306828. doi: 10.1371/journal.pone.0306828 (PMC11326552; doi:10.1371/journal.pone.0306828)
Supplement: S2 Table — (DOCX) [file pone.0306828.s003.docx]

**S2 Table. Factors associated with second breast cancers or locoregional recurrence**

|  | Univariable analysis | |
| --- | --- | --- |
| Variable | Odds ratio | *p* value |
| Age at surgery (y) |  | .10 |
| < 40 | 1.89 (0.88, 4.08) |  |
| ≥ 40 | Reference |  |
| Mammographic breast  density |  | .54 |
| BI-RADS A or B, fatty | Reference |  |
| BI-RADS C or D, dense | 0.82 (0.42, 1.58) |  |
| BRCA mutation status |  | .58 |
| Negative | Reference |  |
| Positive | 1.78 (0.23, 13.49) |  |
| First-degree family history of breast cancer |  | .33 |
| No | Reference |  |
| Yes | 1.70 (0.59, 4.89) |  |
| Stage of primary breast cancer |  | .92 |
| Ductal carcinoma in situ | 0.95 (0.37, 2.48) |  |
| Invasive | Reference |  |
| TNM stage |  | .99 |
| 0 | Reference |  |
| 1 | 1.16 (0.42, 3.17) |  |
| 2 | 1.18 (0.42, 3.33) |  |
| ER and PR status |  | .36 |
| ER- and PR-  negative | 0.66 (0.27, 1.60) |  |
| ER- or PR-  positive | Reference |  |
| HER2 status |  | .47 |
| Negative | Reference |  |
| Positive | 1.33 (0.61, 2.89) |  |
| Type of surgery |  | .50 |
| Breast conserving | 1.27 (0.63, 2.56) |  |
| Mastectomy | Reference |  |
| Adjuvant radiation therapy |  | .86 |
| No | 0.94 (0.47, 1.89) |  |
| Yes | Reference |  |
| Neoadjuvant chemotherapy |  | .20 |
| No | Reference |  |
| Yes | 0.46 (0.14, 1.50) |  |
| Adjuvant chemotherapy |  | .03 |
| No | Reference |  |
| Yes | 2.10 (1.08, 4.09) |  |
| Antihormonal therapy |  | .93 |
| No | 1.03 (0.47, 2.29) |  |
| Yes | Reference |  |
| Note.—Data in parentheses are 95% CIs. ER = estrogen receptor, HER2 = human epidermal growth factor receptor type 2, NA = not applicable, PR = progesterone receptor. | | |
